# Characterization of *BoaCRTISO* Reveals Its Role in Carotenoid Biosynthesis in Chinese Kale

**DOI:** 10.3389/fpls.2021.662684

**Published:** 2021-05-14

**Authors:** Min Jiang, Fen Zhang, Qiao Yuan, Peixing Lin, Hao Zheng, Sha Liang, Yue Jian, Huiying Miao, Huanxiu Li, Qiaomei Wang, Bo Sun

**Affiliations:** ^1^College of Horticulture, Sichuan Agricultural University, Chengdu, China; ^2^Key Laboratory of Horticultural Plant Growth, Development and Quality Improvement, Ministry of Agriculture, Department of Horticulture, Zhejiang University, Hangzhou, China

**Keywords:** *BoaCRTISO*, carotenoid biosynthesis, Chinese kale, color, gene expression, VIGS

## Abstract

Carotenoids are organic pigments that play an important role in both plant coloration and human health; they are a critical subject in molecular breeding due to growing demand for natural molecules in both food and medicine. In this study, we focus upon characterizing *BoaCRTISO*, the carotenoid isomerase gene before the branch of the carotenoid biosynthetic pathway, which is expressed in all organs and developmental stages of Chinese kale, and BoaCRTISO, which is located in the chloroplast. The expression of *BoaCRTISO* is induced by strong light, red and blue combined light, and gibberellic acid treatment, but it is suppressed by darkness and abscisic acid treatment. We obtained *BoaCRTISO*-silenced plants via virus-induced gene silencing technology, and the silence efficiencies ranged from 52 to 77%. The expressions of most carotenoid and chlorophyll biosynthetic genes in *BoaCRTISO*-silenced plants were downregulated, and the contents of carotenoids and chlorophyll were reduced. Meanwhile, *BoaCRTISO*-silenced plants exhibited phenotypes of yellowing leaves and inhibited growth. This functional characterization of *BoaCRTISO* provides insight for the biosynthesis and regulation of carotenoid in Chinese kale.

## Introduction

Chinese kale (*Brassica oleracea* var. *alboglabra*) is a member of Brassicaceae, or the mustard family, which originated in South China and later spread throughout Southeast Asia (Lei et al., 2017). Its main edible parts are the highly nutritious tender leaves and bolting stems (Sun et al., 2018b). Our previous studies have shown that Chinese kale is rich in health-promoting compounds including vitamin C, glucosinolates, and carotenoids (Sun et al., 2012a, b). Carotenoids are the second largest group of natural pigments and are known to play crucial roles in plant color, photosynthesis, and human health (Xiao et al., 2012). The different types and contents of carotenoids lead to the rich and colorful red, yellow, and orange pigments of vegetables, fruits, and flowers such as tomato (Ye et al., 2015), citrus (Kato et al., 2004), watermelon (Lv et al., 2015), and chrysanthemum (Yamagishi et al., 2010). In green plant tissues, carotenoids are also light-harvesting pigments that are closely associated with chlorophylls in the photosynthetic membranes of plants where they protect chlorophyll against photooxidative damage (Guo et al., 2018; Llorente et al., 2020). In humans, carotenoids are necessary to maintain body health. Carotenoids are the precursors of retinoid synthesis and are theorized to act as antioxidants that reduce the risk of macular degeneration (Nisar et al., 2015). It is also reported that carotenoids could treat chronic diseases, such as type 2 diabetes and cardiometabolic diseases, and lower the risk of cancer and cardiovascular disease (Gu et al., 2015; Liu et al., 2015). Given the growing list of human health benefits, horticulturists and others have a deep interest in developing carotenoid-rich food crops through breeding and metabolic engineering. Recent research generally uses molecular breeding methods to enhance carotenoids and to focus on changing biosynthetic pathways to alter metabolic flux (Saini et al., 2014; Kaur et al., 2017), such as golden rice (Beyer et al., 2002) and multivitamin corn (Naqvia et al., 2009).

Currently, the carotenoid biosynthetic pathway in complex and vascular plants has been clarified (Nisar et al., 2015). Several major carotenoids in plants appear in their *trans* form through the activity of isomerases, which are enzymes that function to convert molecules from one isomer to another. For example, carotenoid isomerase (CRTISO) is a key isomerase in the carotenoid biosynthetic pathway, which can catalyze pro-lycopene to lycopene (Pinheiro et al., 2019). In *Arabidopsis* (Park et al., 2002), tomato (Isaacson et al., 2002), and cabbage (Su et al., 2015), the loss of *CRTISO* function could cause the accumulation of pro-lycopene, which delays the greening of *Arabidopsis*, and changes the tomato color from red to orange and the cabbage color from yellow to orange. A *crtiso* mutant in rice produces a so-called zebra phenotype with alternating green and chlorotic crossbands on the mutant leaves (Chai et al., 2011). Besides the aesthetic effect on plant color, *CRTISO* also has a significant functional effect on photosynthetic efficiency. For instance, chlorophyll accumulation during photomorphogenesis was delayed markedly in a *ccr2* (*Atcrtiso*) mutant (Park et al., 2002), while the photosynthetic rate of yellow leaf sectors produced from a *crtiso* mutant in rice was approximately 27–42% of that of the wild-type unaffected leaves (Chai et al., 2011). In addition, carotenoid contents and related gene expressions can be regulated by exogenous phytohormone and light treatments in most plants (Divya et al., 2018). The exogenous methyl jasmonate (MeJA) spray treatment significantly enhanced the accumulation of lycopene in tomato (Liu et al., 2012) and other carotenoids in leafy vegetables (Divya et al., 2014; Saini et al., 2014), and abscisic acid (ABA) also had the similar effect in tomato (Sun et al., 2012), whereas gibberellic acid (GA_3_) treatment had a marked effect on preventing the carotenoid accumulation in Navelate oranges (Rodrigo and Zacarias, 2007). It has also been found that the expression levels of *BoaPDS1* and *BoaPDS2* genes in Chinese kale were both regulated by red and blue combined light, and salicylic acid treatments (Sun et al., 2019). We suggest that these abiotic environmental conditions could regulate carotenoid biosynthetic pathways through *CRTISO*.

At present, the development of Chinese kale in China is relatively slow compared with other brassica vegetables. One of the key limiting reasons is that its color is not rich enough, and most of the germplasm resources are green (Lei et al., 2017), which makes it very difficult to breed new varieties with other colors via crossbreeding. In this study, we investigated the expression patterns of *BoaCRTISO* under different conditions; and we obtained *BoaCRTISO*-silenced plants using virus-induced gene silencing (VIGS) technology, and then we analyzed changes in the phenotype, carotenoid contents, and the related biosynthetic gene expressions of *BoaCRTISO*-silenced plants. Our findings allow a better understanding of the function of *BoaCRTISO* in regulating the carotenoid contents and coloring in Chinese kale, and this work will help in the creation of new varieties of Chinese kale from the perspective of molecular breeding.

## Materials and Methods

### Plant Materials

In this study, we used the white-flower Chinese kale, *Brassica oleracea* var. *alboglabra* “Sijicutiao.” The plants were grown in trays containing a mixture of peat and vermiculite (3:1) in an incubator with a light intensity of 80 μmol m^–2^ s^–1^, a temperature of 25/20°C (day/night), a 12/12-h (day/night) light cycle, and 75% humidity. Seedlings with five to six true leaves were transplanted to the greenhouse of Sichuan Agricultural University for cultivation. Fertilizer and water were applied as needed.

Chinese kale materials were sampled at different developmental stages (germinating seeds, cotyledons, fifth to sixth true leaves, and mature leaves), as were organs at different stages of maturation (roots, bolting stems, petioles, leaves, inflorescence, fruit pod, and young fruit), and floral organs at the flower buds stage and the opening flowers stage (sepals, petals, stamens, and pistils). The materials were frozen by liquid nitrogen and stored at −80°C for subsequent studies (Sun et al., 2019).

### Light and Phytohormone Treatments

The 30-day-old Chinese kale seedlings, raised in identical growth conditions, were selected for light intensity, light quality, and phytohormone treatments (Sun et al., 2019). The light quality treatments were divided into red light (R:B = 10:0), blue light (R:B = 0:10), and red and blue combined light (R:B = 5:5) treatments, while white light treatment was used as a control. Light intensity treatments were divided into darkness (0 μmol m^–2^ s^–1^), weak light (40 μmol m^–2^ s^–1^), and strong light (120 μmol m^–2^ s^–1^) groups; the control group was exposed to white light (80 μmol m^–2^ s^–1^). Phytohormones were sprayed on the leaf surface with 5 μM of GA_3_ and 0.5 μM of ABA, and distilled water was used as control. When the leaves ceased dripping, the plants were moved into an artificial intelligence light incubator. In addition, 100 μM of MeJA was used to fumigate the Chinese kale seedlings in a transparent and closed container. The cultural conditions of the control and treated Chinese kale seedlings were as described in section “Plant Materials.” The fifth to sixth true leaves were sampled at 0, 1, 3, 6, 12, 24, 48, and 72 h after each treatment, and the samples were immediately frozen in liquid nitrogen and stored at −80°C until RNA extraction.

### RNA Extraction and qPCR Analysis

Total RNA was extracted from Chinese kale using a cetyl trimethylammonium bromide (CTAB) method (Chen et al., 2011). The well-integrated total RNA was used for cDNA synthesis using the PrimeScript^TM^ 1st Strand cDNA Synthesis Kit (TAKARA, Japan). The qPCR primers for carotenoid and chlorophyll biosynthetic genes in Chinese kale were synthesized based on the qPCR primer database^1^, and β*-actin* (Büchert et al., 2011) was used as the reference gene (Supplementary Table 1). The qPCR was performed referring to the TB Green Premix Ex Taq II (Tli RNaseH Plus) (TAKARA, Japan) kit instructions by using the Bio-Rad iCycler thermocycler (Bio-Rad, United States). Gene expression analysis was performed using the 2^–ΔΔCT^ method (Livak and Schmittgen, 2001).

### Molecular Cloning and Sequence Analysis

According to the sequences of the *CRTISO* gene and promoters of homologous species such as cabbage and Chinese cabbage published by Brassica database (BRAD), specific primers for the *BoaCRTISO* gene and promoters were designed (Supplementary Table 1). The method of gene cloning refers to Sun et al. (2018b). The CRTISO amino acid sequence of other species was downloaded from the National Center for Biotechnology Information (NCBI) and subjected to multiple sequence alignment using DNAMAN software (Lynnon Biosoft, United States). The phylogenetic tree was generated using the neighbor-joining method by MEGA 6.0 software (Tamura et al., 2013). The *cis*-acting elements on the promoter sequences of *BoaCRTISO* were predicted using the PlantCARE online software^2^.

### Subcellular Localization

Subcellular localization of BoaCRTISO was performed using the methods described by Sun et al. (2018a). The complete coding sequence (CDS) of *BoaCRTISO* was amplified using the primers BoaCRTISO-GFP-F/R (Supplementary Table 1), in which a *Bam*HI site at the 5′-end and a *Spe*I site at the 3′-end of the gene were incorporated. The BoaCRTISO and the pC2300-35S-eGFP plasmid digested with *Bam*HI and *Spe*I were mixed to generate pC2300-35S-*BoaCRTISO*-eGFP plasmid. Chinese kale mesophyll protoplasts were isolated and purified, and then the pC2300-35S-*BoaCRTISO*-eGFP and pC2300-35S-eGFP plasmids were transformed into Chinese kale protoplasts. The protoplasts expressing green fluorescent protein (GFP)-fusion protein were observed, and images were captured using a BX-51 fluorescence microscope equipped with a DP70 camera (Olympus, Japan).

### Virus-Induced Gene Silencing of BoaCRTISO

A turnip yellow mosaic virus (TYMV)-induced gene silencing system was used to functionally characterize *BoaCRTISO* according to the previously described methods (Muntha et al., 2018). The pTY vector was digested with *Sna*BI, and the resulting linearized vector was analyzed by gel electrophoresis to confirm specificity. The linearized pTY vector and the annealed 80-base-pair-specific nucleotide of *BoaCRTISO* were ligated using T4 ligase to form a pTY-BoaCRTISO vector. The amplification of a *TYMV-CP* gene of the expected size (520 nt) was used to identify positive clones. Details of the pTY-CP F/R primer pairs are provided in Supplementary Table 1. For the virus infiltration, 5 μg of purified pTY-S carrying the target gene was diluted in 25 μl of double-distilled H_2_O, which was then used to infiltrate two to four leaves of each plant. The infiltration was repeated every 5 days for 20 days, for a total of four interventions. The control plants were infiltrated with water, and the plants infiltrated with an empty pTY-S vector were used as the reference. Infiltrated plants were incubated in a growth chamber set at 22/20°C (day/night) with an 8/16-h (light/dark) cycle. One week after the last infiltration, plant phenotype analysis was performed, and the newly grown leaves were sampled for carotenoid composition, content, and related gene expression analysis.

### Color

Color analysis of the transgenic plants was conducted using an NR110 chroma meter (3 nh, China). Three positions on the sampled leaves of each infiltrated plant were randomly selected, and the color values *L*^∗^, *a*^∗^, and *b*^∗^ were obtained.

### Determination of Carotenoid and Chlorophyll Composition and Contents

Carotenoid and chlorophyll concentrations were determined using the methods of Sun et al. (2021) with a slight modification. Approximately 200 mg of leaves was ground and extracted with 25 ml of acetone. The samples were sonicated for 20 min and then centrifuged at 4,000 *g* at room temperature for 5 min. The supernatant was filtered through 0.22-μm cellulose acetate filters and analyzed by high-performance liquid chromatography (HPLC). HPLC analysis of carotenoids and chlorophyll was carried out using an Agilent 1260 instrument with a VWD detector (Agilent, United States). Samples (10 μl) were separated at 30°C on a Waters C18 column (150 × 3.9-mm id; 4-μm particle size) using isopropanol and 80% acetonitrile–water at a flow rate of 0.5 ml min^–1^. Absorbance was detected at 448 and 428 nm.

### Statistical Analysis

All the results are shown as the means of three replicates. Statistical analysis was performed using SPSS version 18 (SPSS Inc., United States). The data were analyzed using one-way analysis of variance (ANOVA), and differences were compared using the least significant difference (LSD) test at a significance level of 0.05.

## Results

### Isolation and Characterization of the BoaCRTISO

**FIGURE 1:**
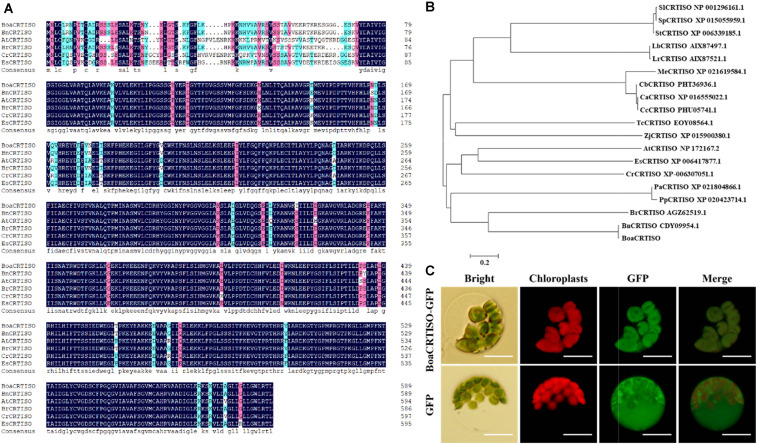
Sequence characteristics of BoaCRTISO. **(A)** Alignment of the protein sequence of BoaCRTISO with selected homologs. The alignment was performed using DNAMAN software. The amino acids with 100% identity are shown with a black background, those with ≥75% identity are shown in red, and those with ≥50% identity are shown in blue. The species and their accession numbers in GenBank [*Brassica napus* (Bn): BnCRTISO (CDY09954.1), *Brassica rapa* (Br): BrCRTISO (AGZ62519.1), *Arabidopsis thaliana* (At): AtCRTISO (NP_172167.2), *Capsella rubella* (Cr): CrCRTISO (XP_006307051.1), and *Eutrema salsugineum* (Es): EsCRTISO (XP_006417877.1)] are listed here. **(B)** Phylogenetic analysis of BoaCRTISO and selected CRTISO from other plant species. The phylogenetic tree was generated using the neighbor-joining method by MEGA 6.0 software. The bar indicates an evolutionary distance of 0.2%. The species [*B. napus* (Bn), *B. rapa* (Br), *A. thaliana* (At), *C. rubella* (Cr), *E. salsugineum* (Es), *Prunus persica* (Pp), *Lycium barbarum* (Lb), *Lycium ruthenicum* (Lr), *Prunus avium* (Pa), *Manihot esculenta* (Me), *Ziziphus jujube* (Zj), *Solanum lycopersicum* (Sl), *Theobroma cacao* (Tc), *Solanum pennellii* (Sp), *Solanum tuberosum* (St), *Capsicum baccatum* (Cb), *Capsicum annuum* (Ca), and *Capsicum chinense* (Cc)] are listed here. **(C)** Subcellular localization of the BoaCRTISO-GFP fusion protein in Chinese kale protoplasts. Free green fluorescent protein served as a control. Bars = 30 μm.

Analysis of the *Brassica oleracea* genome data revealed that the *CRTISO* gene has only one member. The results of cloning of *BoaCRTISO* also showed that there is only one *BoaCRTISO* in Chinese kale. The CDS of the *BoaCRTISO* was cloned with sequence lengths of 1,773 bp (GenBank accession MN810158), which encodes a 590-amino-acid protein. Alignment results showed that the changes in amino acid sequences of CRTISO among different species mainly occurred at the N-terminus (Figure 1A). A phylogenetic analysis showed that BoaCRTISO clustered with other *Cruciferae* CRTISO (Figure 1B), which indicates that CRTISO is highly conserved in cruciferous plants. Moreover, a construct encoding BoaCRTISO fused to GFP was transformed into Chinese kale protoplasts, and strong fluorescence from GFP-BoaCRTISO was detected in the chloroplast (Figure 1C), which clearly demonstrates that BoaCRTISO localizes to the chloroplast.

### Temporal and Spatial Expressions of BoaCRTISO

**FIGURE 2:**
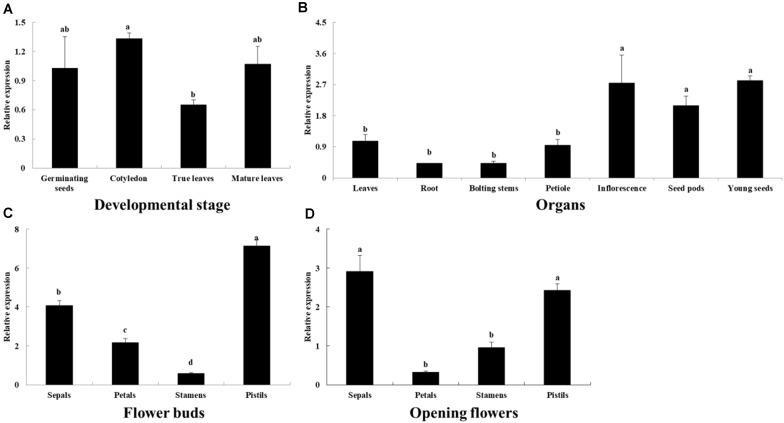
Expression levels of *BoaCRTISO* in different developmental stages: **(A)** organs, **(B)** flower organs in flower buds stage **(C)**, and opening flowers stage **(D)** of Chinese kale. The *BoaCRTISO* expression of germinating seeds was set as 1. The samples were a mixture from three individual plants. Data are expressed as mean ± standard deviation. The same letter in the same histogram indicates that there is no significant difference between the values tested by least significant difference (LSD) (*p* < 0.05).

The *BoaCRTISO* gene was expressed in all developmental stages and organs (Figure 2). During the development of Chinese kale, the highest level of *BoaCRTISO* expression was in the cotyledon stage, followed by the germination and maturity stages, and it was lowest in the true leaves stage (Figure 2A). In different organs during the mature period, the expression levels of *BoaCRTISO* in inflorescences, seed pods, and young seeds were relatively high and were notably more than twice those of other organs (Figure 2B). During the flower buds and opening flowers stages, the expression levels of *BoaCRTISO* in both pistils and sepals were more than twice those observed in petals and stamens. Compared with the flower buds stage, the expression of *BoaCRTISO* in all flower organs was significantly downregulated at the opening flowers stage, except for stamens.

### Light and Phytohormone Treatments Affected BoaCRTISO Expression

**FIGURE 3:**
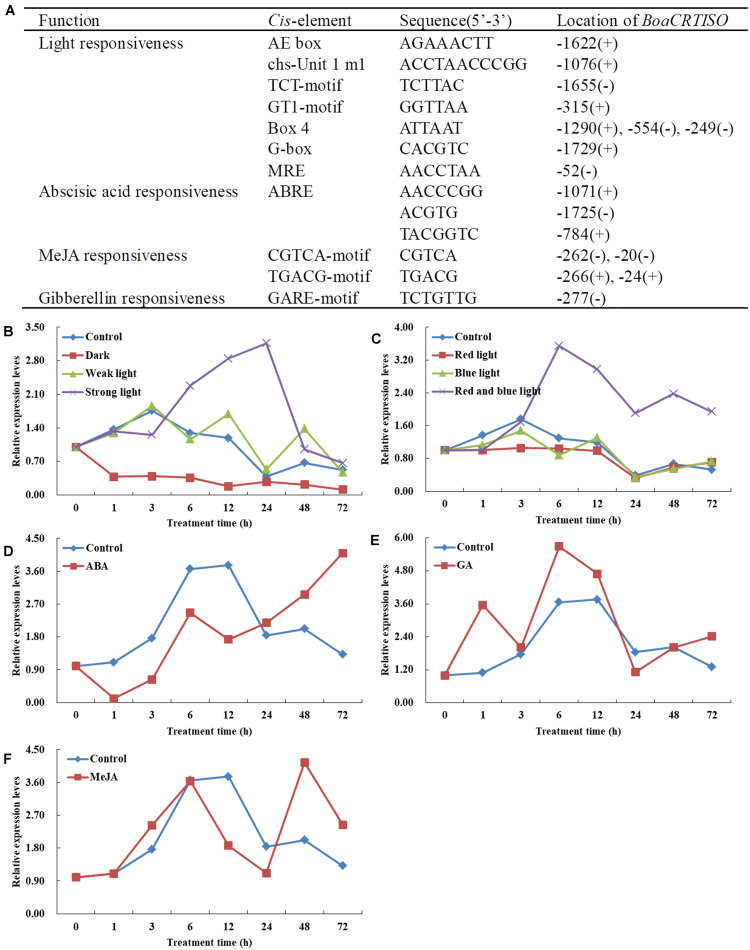
Response of *BoaCRTISO* to different exogenous treatments. **(A)** *Cis*-acting elements respond to light and phytohormone in the promoter regions of *BoaCRTISO*. The + and − in brackets represent sense strand and antisense strand, respectively. **(B)** Expression levels of *BoaCRTISO* after treatments with darkness, weak light, and strong light. **(C)** Expression levels of *BoaCRTISO* after treatments with red light, blue light, and combined red and blue lights. **(D)** Expression level of *BoaCRTISO* after abscisic acid (ABA) treatment. **(E)** Expression level of *BoaCRTISO* after GA_3_ treatment. **(F)** Expression level of *BoaCRTISO* after methyl jasmonate (MeJA) treatment.

The promoter of the *BoaCRTISO* from Chinese kale leaves was cloned with sequence lengths of 1,789 bp (GenBank accession MN810159). The *cis*-elements of *BoaCRTISO* promoter mainly contained light- and phytohormone-responsive elements (Figure 3A). The light-responsive elements included AE-box, Box 4, TCT-motif, chs-Unit 1 m 1, G-box, GT1-motif, and MRE, while phytohormone-responsive elements included ABA-responsive element, MeJA response elements (CGTCA-motif and TGACG-motif), and GA response element (GARE-motif).

Under the treatment of different light intensities, it can be seen that the expression of *BoaCRTISO* is significantly induced by strong light and significantly inhibited in the dark, while weak light has little effect on it (Figure 3B and Supplementary Table 2). Under the combined red and blue light treatments, the expression level of *BoaCRTISO* was significantly higher than that of the control group after 3 h, and its peak value was about three times that of the control group at 6 h. Compared with that of the control group, the expression of *BoaCRTISO* was significantly lower under red light treatment, but there was no significant difference under blue light treatment (Figure 3C and Supplementary Table 2). Under ABA treatment, the expression level of *BoaCRTISO* was inhibited before 24 h, but the inhibition gradually weakened afterward (Figure 3D and Supplementary Table 2). Under GA_3_ treatment, the expression level of *BoaCRTISO* was significantly induced (Figure 3E and Supplementary Table 2), while the expression level of *BoaCRTISO* had no obvious regularity under MeJA treatment (Figure 3F and Supplementary Table 2).

### BoaCRTISO Silencing Affected the Color and Inhibited the Growth of Chinese Kale

**FIGURE 4:**
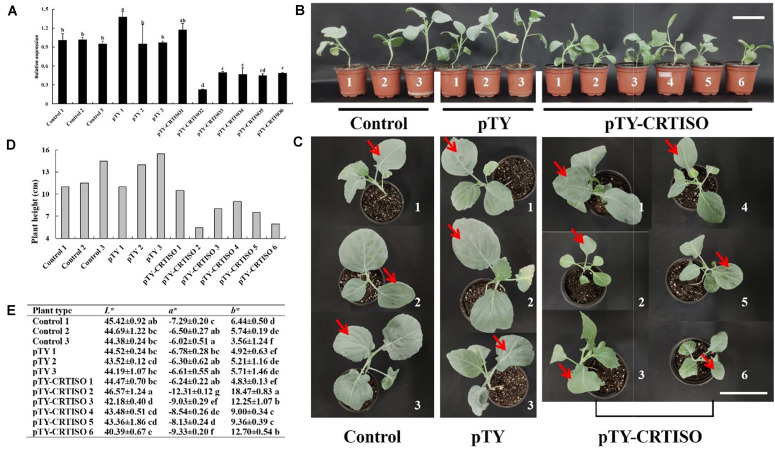
*BoaCRTISO* silencing affects the color and growth of Chinese kale. **(A)** Expressions of *BoaCRTISO* in control, pTY, and pTY-BoaCRTISO plants. Data are expressed as mean ± standard deviation. The same letter indicates that there is no significant difference between the values tested by least significant difference (LSD) (*p* < 0.05). **(B)** Front view of control, pTY, and pTY-BoaCRTISO plants. Bar = 10 cm. **(C)** Top view of control, pTY, and pTY-BoaCRTISO plants. The red arrow points to the sampled leaves. Bar = 10 cm. **(D)** Plant heights of control, pTY, and pTY-BoaCRTISO plants at 1 week after the last infiltration. **(E)** The color parameters of control, pTY, and pTY-BoaCRTISO plants at 1 week after the last infiltration. Data are expressed as a mean ± SD. The same letter in the same column means no significant differences among values (*p* < 0.05) according to a least significant difference (LSD) test.

The expressions of *BoaCRTISO* in all pTY-BoaCRTISO plants were significantly reduced compared with control and the pTY plants, except for pTY-BoaCRTISO 1 plant (Figure 4A). Then, we observed that the pTY-BoaCRTISO plants were yellow-green compared with the dark-green of the control and the pTY plants, except for the pTY-BoaCRTISO 1 plant (Figures 4B,C). The changes of color between the pTY-BoaCRTISO plants, control, and pTY plants were then analyzed (Figure 4D). The yellow-blue value *b*^∗^ of pTY-BoaCRTISO 2–6 plants were significantly higher than that of the control and pTY plants, which indicated that pTY-BoaCRTISO 2–6 plants were yellowing. The *b*^∗^ value of the pTY-BoaCRTISO 2 plant with the most remarkable color change was 18, which was more than three times of that of the control and pTY plants. The red-green value *a*^∗^ of pTY-BoaCRTISO 2–6 plants was significantly lower than that of control and pTY plants. The *L*^∗^ value is similar among different groups. However, the *L*^∗^, *a*^∗^, and *b*^∗^ values of pTY-BoaCRTISO 1 plant were all similar to those of control and pTY plants. In addition, the growth of pTY-BoaCRTISO plants were significantly inhibited, and the plant heights were significantly reduced compared with control and pTY plants, except for pTY-BoaCRTISO 1 plant (Figures 4B,E). Therefore, control 1, pTY 3, and pTY-BoaCRTISO 2, 5, and 6 with typical phenotypes and relatively high silencing efficiencies were chosen for subsequent analyses.

### BoaCRTISO Silencing Reduced Carotenoids and Chlorophyll Accumulation in Chinese Kale

**FIGURE 5:**
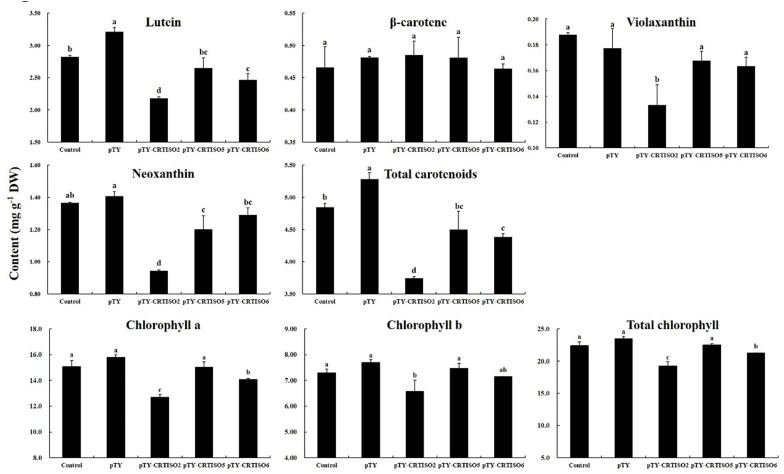
Carotenoid composition and contents in control, pTY, and pTY-BoaCRTISO plants. Samples of leaves were taken from control, pTY, and pTY-BoaCRTISO plants at 1 week after the last infiltration. Data are expressed as mean ± standard deviation. The same letter in the same histogram indicates that there is no significant difference between the values tested by least significant difference (LSD) (*p* < 0.05).

After infection with the pTY-BoaCRTISO plasmid, the expression levels of *CRTISO* of pTY-BoaCRTISO 2, 5, and 6 were significantly reduced and were 23%, 45%, and 48% of the control plants, while the silencing efficiencies were 77%, 55%, and 52%, respectively (Supplementary Figure 1). Next, the total and individual carotenoid contents in pTY-BoaCRTISO plants were analyzed, and we found that their contents were decreased compared with control and pTY plants, except that there was no significant change in β-carotene (Figure 5). Specifically, lutein, violaxanthin, and neoxanthin in pTY-BoaCRTISO 2 plant were only approximately 70% of the control. Moreover, the contents of lutein, neoxanthin, and violaxanthin in pTY-BoaCRTISO 5 and 6 plants were all reduced, but only significantly so in neoxanthin and lutein. Since lutein content accounted for more than 60% of the total carotenoid levels in Chinese kale, the total carotenoid contents in pTY-BoaCRTISO 2 and 6 plants were significantly reduced when their lutein contents were significantly inhibited.

Chlorophyll, as a pigment closely related to carotenoids, has also been analyzed for its content. The individual and total chlorophyll of the pTY-BoaCRTISO 2 plant with the lowest *BoaCRTISO* expression decreased significantly. The changes in chlorophyll of pTY-BoaCRTISO 5 and 6 plants whose expression level of *BoaCRTISO* decreased by half were different. Chlorophyll *a* and total chlorophyll in pTY-BoaCRTISO 6 plants decreased significantly, whereas the individual and total chlorophylls of pTY-BoaCRTISO 5 plants did not change significantly.

### BoaCRTISO Silencing Suppressed the Expression of Carotenoid and Chlorophyll Biosynthetic Genes

**FIGURE 6:**
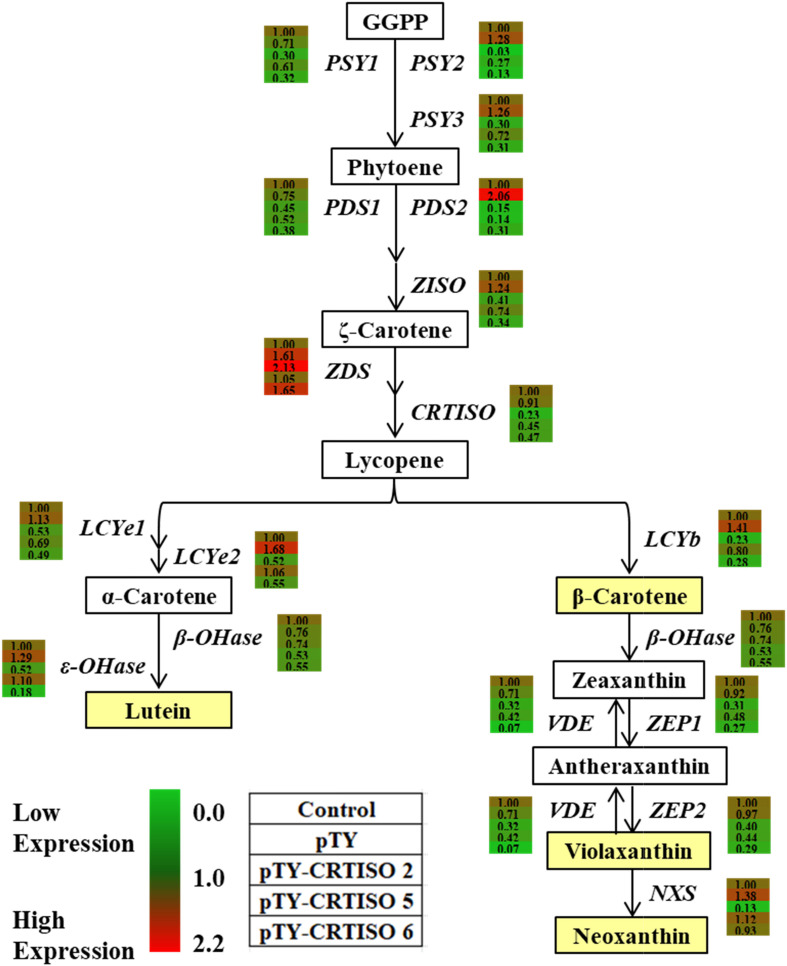
Heat map of carotenoid biosynthetic gene expressions in control, pTY, and pTY-BoaCRTISO plants. Samples of leaves were taken from control, pTY, and pTY-BoaCRTISO plants at 1 week after the last infiltration. GGPP, geranylgeranyl diphosphate; PSY, phytoene synthase; PDS, phytoene desaturase; ZDS, ζ-carotene desaturase; Z-ISO, ζ-carotene isomerase; CRTISO, carotenoid isomerase; LCYe, lycopene ε-cyclase; LCYb, lycopene β-cyclase; ε-OHase, ε-carotene hydroxylase; β-OHase, β-carotene hydroxylase; VDE, violaxanthin de-epoxidase; ZEP, zeaxanthin epoxidase; NXS, neoxanthin synthase.

To investigate whether *BoaCRTISO* affects carotenoid accumulation by regulating the transcription of carotenoid biosynthetic genes, their expressions in the pTY-BoaCRTISO plants were analyzed by using RT-qPCR (Figure 6 and Supplementary Figure 1). We found that the expression levels of all carotenoid biosynthetic genes in the pTY-BoaCRTISO 2 plant were significantly downregulated except *ZDS*, which was significantly upregulated, and β*-OHase*, which was unchanged. Regarding pTY-BoaCRTISO 5 and 6 plants, the expressions of most of their carotenoid biosynthetic genes were also downregulated, while the expressions of *ZDS*, *LCYe2*, and ε*-OHase* genes of pTY-BoaCRTISO 5 plant did not change significantly, and the expressions of *ZDS* and slightly increased while *NXS* did not change in the pTY-BoaCRTISO 6 plant.

In pTY-BoaCRTISO plants, chlorophyll, as the main contributor to the color of the leaves of Chinese kale, content decreased significantly, which made us pay attention to the expression of chlorophyll biosynthetic genes. The results indicated that the expression levels of the most chlorophyll biosynthetic genes detected were significantly decreased in the pTY-BoaCRTISO plants (Supplementary Figure 2). These findings suggest that the downregulation of *BoaCRTISO* expression has an inhibitory effect on the entire carotenoid and chlorophyll biosynthetic pathway in Chinese kale.

## Discussion

The short-duration conventional VIGS has many advantages, including easy and rapid results, unnecessary use of stable plant transformation, and the ability to apply multiple copies or family members (Senthil-Kumar and Mysore, 2011). In this study, the short, inverted repeat coding for *BoaCRTISO* was inserted into the VIGS system of TYMV to silence the targeted gene, and the resulting *BoaCRTISO*-silenced plants were successfully obtained with a silencing efficiency of 52–77%. The efficiency of VIGS can be affected by multiple factors, such as crop species and virus types (Senthil-Kumar and Mysore, 2011). Several previous studies indicated that the silencing efficiencies of *Brassica juncea*, *Catharanthus roseus*, and strawberry were 90% (Muntha et al., 2018), 70% (Kumar et al., 2020), and 45% (Xie et al., 2020), respectively, which demonstrates the varying degrees of effectiveness across species. We have previously used tobacco rattle virus-mediated VIGS technology for gene silencing in Chinese kale; however, the efficiency of gene silencing was poor (data available upon request). Therefore, we suggest selecting an appropriate virus type when conducting VIGS assays for different crop species.

The role of carotenoids in plant coloration is well studied, and the biosynthetic pathways have been generally described. In this study, it was found that the leaves of Chinese kale color turned yellow and the *b*^∗^ value increased in pTY-BoaCRTISO-treated plants that silenced *BoaCRTISO* expression. This result is consistent with several previous results but inconsistent with a few others. Our findings are similar to those on seedlings of melons with EMS mutagenesis, which leads to CRTISO being downregulated, or silenced, which appear yellow-green (Galpaz et al., 2013). In a separate study, the leaves showed a yellow-green zebra phenotype in a *crtiso* mutant in rice (Chai et al., 2011). However, the mutation of *CRTISO* in tomatoes changes the fruit phenotype to orange (Isaacson et al., 2002). These findings indicate that the phenotypes of leaves and other organs are expressed differently according to species after *CRTISO* is silenced. Besides, after the *BoaCRTISO* gene was target-edited in Chinese kale, the plant showed a distinct yellow color, although the gene expression level was similar to that of the VIGS plants (Sun et al., 2020). This shows that stable plant transformation can make plants show a more obvious phenotype than transient VIGS technology. In addition to leaves and fruits, color changes of the floral organs are also related to *BoaCRTISO* expression. We found that expression of *BoaCRTISO* in the flower buds stage of Chinese kale was significantly higher than that in the flowering stage, and this finding was contrary to the results of studies on lilies (Yamagishi et al., 2010) and chrysanthemums (Kishimoto and Ohmiya, 2006). This is likely because the Chinese kale flowering process is one of carotenoid degradation, and the opposite process occurs in the chrysanthemum and lily.

After determining that *BoaCRTISO* partial suppression can yellow Chinese kale leaves, we further explored the relationship among its phenotype, gene expression, and carotenoid contents. Prior work showed that the “Micro Tom” tomato was silenced using an inverted repeat of a fragment from the *Citrus CRTISO* that resulted in an overall decrease in transcript accumulation of all genes from the carotenoid biosynthetic pathway (Pinheiro et al., 2019), whereas downregulation of *CRTISO* expression in tomato (Isaacson et al., 2002) and cabbage (Su et al., 2015) can lead to upregulation of upstream gene expression and downregulation of downstream gene expression in the carotenoid biosynthetic pathway, causing the accumulation of pro-lycopene. In our study, we found that after *BoaCRTISO* expression was downregulated, almost all carotenoid biosynthetic gene expressions in Chinese kale were also downregulated; this finding is consistent with results reported on citrus (Pinheiro et al., 2019) but inconsistent with that known from tomato (Isaacson et al., 2002) and cabbage (Su et al., 2015). Lutein is the primary component of carotenoids in Chinese kale, while prolycopene and lycopene are not detected. Therefore, the downregulation of *BoaCRTISO* expression led to the overall decline of carotenoid contents rather than the accumulation of prolycopene, as has been reported in other species. These results suggest that *CRTISO* might have variable functions in different species and that it could regulate the entire suite of carotenoid biosynthetic genes and carotenoid contents in Chinese kale.

We also found that *BoaCRTISO* expression can be regulated by exogenous light and hormone treatments. In our study, *BoaCRTISO* expression was induced under strong light, but it was inhibited in darkness compared with the control plants; this indicates that *CRTISO* is a light-inducible gene, which is consistent with the expression changes of citrus *CRTISO* under light and dark conditions (Gao et al., 2011). A few previous reports suggested that mixed (red and blue) or white lights could stimulate carotenoid contents in plants in comparison with applications of only red or only blue light (Tamura et al., 2013; Mastropasqua et al., 2020). Similarly, we found that the red and blue combination light treatment significantly induced *BoaCRTISO* expression compared with applications of only red, blue, or white light. In addition to light, *BoaCRTISO* expression was also regulated by ABA, GA_3_, and MeJA exogenous treatments. After ABA treatment, we observed that *BoaCRTISO* expression decreases significantly in the early stage; this may be due to the effect of feedback inhibition where endogenous ABA decreased because the content of exogenous ABA increased, and then the biosynthesis of carotenoids (which are an ABA precursor) decreased accordingly. In a similar fashion, it has been reported that gibberellin synthesis is inhibited after overexpression of a lycopene β-cyclase gene in tobacco, which indicates that gibberellin could negatively regulate carotenoid biosynthesis (Moreno et al., 2016). However, we found that *BoaCRTISO* expression was positively regulated by GA_3_ treatment, which may be because the tested carotenoid biosynthetic genes differed in the previous study. In addition, MeJA has a dosage effect of high concentration inhibition and low concentration promotion on the regulation of carotenoids in tomato (Liu et al., 2012), which is consistent with our results. These results suggest that external environmental changes could be manipulated to regulate carotenoid biosynthesis.

Meanwhile, CRTISO plays an important role in photosynthesis and plant growth (Xie et al., 2019). In rice, the reduced photosynthetic rate of *crtiso* mutant leaves proved that *CRTISO* has an effect on photosynthesis (Chai et al., 2011). In our study, the expression of *BoaCRTISO* in leaves was significantly higher than that in root organs, and the subcellular localization of BoaCRTISO was in the chloroplast; this is a key organelle for photosynthesis, and the localization we observed may have occurred because leaves are the main photosynthetic organs and require more carotenoids than other plant tissues (Nisar et al., 2015). These results proved that *BoaCRTISO* plays an important role in photosynthesis by Chinese kale. In addition, the temporal and spatial expression patterns of *BoaCRTISO* were constitutive, and the heights of pTY-BoaCRTISO 2, 5, and 6 plants were significantly reduced, which indicates that *BoaCRTISO* is a necessary gene for the growth and development of Chinese kale. When the expression of carotenoid biosynthetic genes decreased, the growth and development of fleshy roots of carrots were also restricted (Flores-Ortiz et al., 2020), which is consistent with our results. In addition, the chlorophyll content and biosynthetic gene expressions in pTY-BoaCRTISO plants were significantly reduced, which would also affect photosynthesis to inhibit plant growth. This suggested that *BoaCRTISO* may affect plant growth by directly or indirectly affecting photosynthesis; nevertheless, the specific mechanism needs further study.

In summary, the carotenoid isomerase gene *BoaCRTISO* and promoter of Chinese kale were cloned, and BoaCRTISO was located in the chloroplast via a subcellular localization assay. The expression of *BoaCRTISO* was detected in all growth and development periods, and organs, of Chinese kale. VIGS-mediated *BoaCRTISO* silencing suppressed the expression of carotenoid and chlorophyll biosynthetic genes, decreased carotenoid and chlorophyll contents, yellowed Chinese kale, and inhibited Chinese kale growth. In addition, *BoaCRTISO* expression can be induced by strong light, combined red and blue light, and GA_3_ treatments but inhibited by darkness and ABA treatments. These findings collectively mean that *BoaCRTISO* is a viable gene to target for improvements of the appearance and nutritional quality of Chinese kale for the betterment of human health.

## Data Availability Statement

The original contributions presented in the study are included in the article/Supplementary Material, further inquiries can be directed to the corresponding authors.

## Author Contributions

MJ: investigation, data curation, and writing – original draft. FZ: methodology and writing – original draft. QY and PL: data curation. HZ, SL, and YJ: investigation. HM: methodology and data curation. QW and HL: conceptualization, funding acquisition, and writing – review and editing. BS: conceptualization, funding acquisition, writing – review and editing, and supervision. All authors review and editing.

## Conflict of Interest

The authors declare that the research was conducted in the absence of any commercial or financial relationships that could be construed as a potential conflict of interest.

## Footnotes

1 https://biodb.swu.edu.cn/qprimerdb/

2 http://bioinformatics.psb.ugent.be/webtools/plantcare/html/

## Supplementary Material

Supplementary Figure 1 Expressions of carotenoid biosynthetic genes in control, pTY, and pTY-BoaCRTISO plants. Samples of leaves were taken from control, pTY, and pTY-BoaCRTISO plants at 1 week after the last infiltration. The carotenoid biosynthetic gene expressions were calculated based on the respective expression of the respective genes in control plant. Data are expressed as mean ± standard deviation. The same letter in the same histogram indicates that there is no significant difference between the values tested by LSD (*p* < 0.05).

Supplementary Figure 2 Expressions of chlorophyll biosynthetic genes in control, pTY, and pTY-BoaCRTISO plants. Samples of leaves were taken from control, pTY, and pTY-BoaCRTISO plants at 1 week after the last infiltration. The chlorophyll biosynthetic gene expressions were calculated based on the respective expression of the respective genes in control plant. Data are expressed as mean ± standard deviation. The same letter in the same histogram indicates that there is no significant difference between the values tested by LSD (*p* < 0.05). ALAD, 5-aminolevulinic acid dehydratase; HemE1, glutamyl tRNA reductase; Chl, magnesium-chelatase; CS, chlorophyll synthase.
