# Supplementary material for: Characterization of BoaCRTISO Reveals Its Role in Carotenoid Biosynthesis in Chinese Kale
Source: Front Plant Sci. 2021 May 14;12:662684. doi: 10.3389/fpls.2021.662684 (PMC8160315; doi:10.3389/fpls.2021.662684)
Supplement: Supplementary Figure 1 — Expressions of carotenoid biosynthetic genes in control, pTY, and pTY-BoaCRTISO plants. Samples of leaves were taken from control, pTY, and pTY-BoaCRTISO plants at 1 week after the last infiltration. The carotenoid biosynthetic gene expressions were calculated based on the respective expression of the respective genes in control plant. Data are expressed as mean ± standard deviation. The same letter in the same histogram indicates that there is no significant difference between the values tested by LSD (p < 0.05). [file Data_Sheet_1.PDF]

Supporting information

Characterization of *BoaCRTISO* reveals its role in carotenoid biosynthesis in Chinese kale

Running title: Function of carotenoid isomerase gene

Min Jiang[1,†], Fen Zhang[1,†], Qiao Yuan[1], Peixing Lin[1], Hao Zheng[1], Sha Liang[1], Yue Jian[1], Huiying Miao[2],Huanxiu Li[1], Qiaomei Wang[2,*], and Bo Sun[1,*]

[1]College of Horticulture, Sichuan Agricultural University, Chengdu 611130, China

[2]Key Laboratory of Horticultural Plant Growth, Development and Quality Improvement, Ministry of Agriculture, Department of Horticulture, Zhejiang University, Hangzhou 310058, China

[†] These authors contributed equally.

***Author for correspondence:**

Qiaomei Wang

Tel: 86-571-88982278; Fax: 86-571-88982278; Email: qmwang@zju.edu.cn

Bo Sun

Tel: 86-28-86291941; Fax: 86-28-86291840; Email: bsun@sicau.edu.cn

Supplementary Figure 1

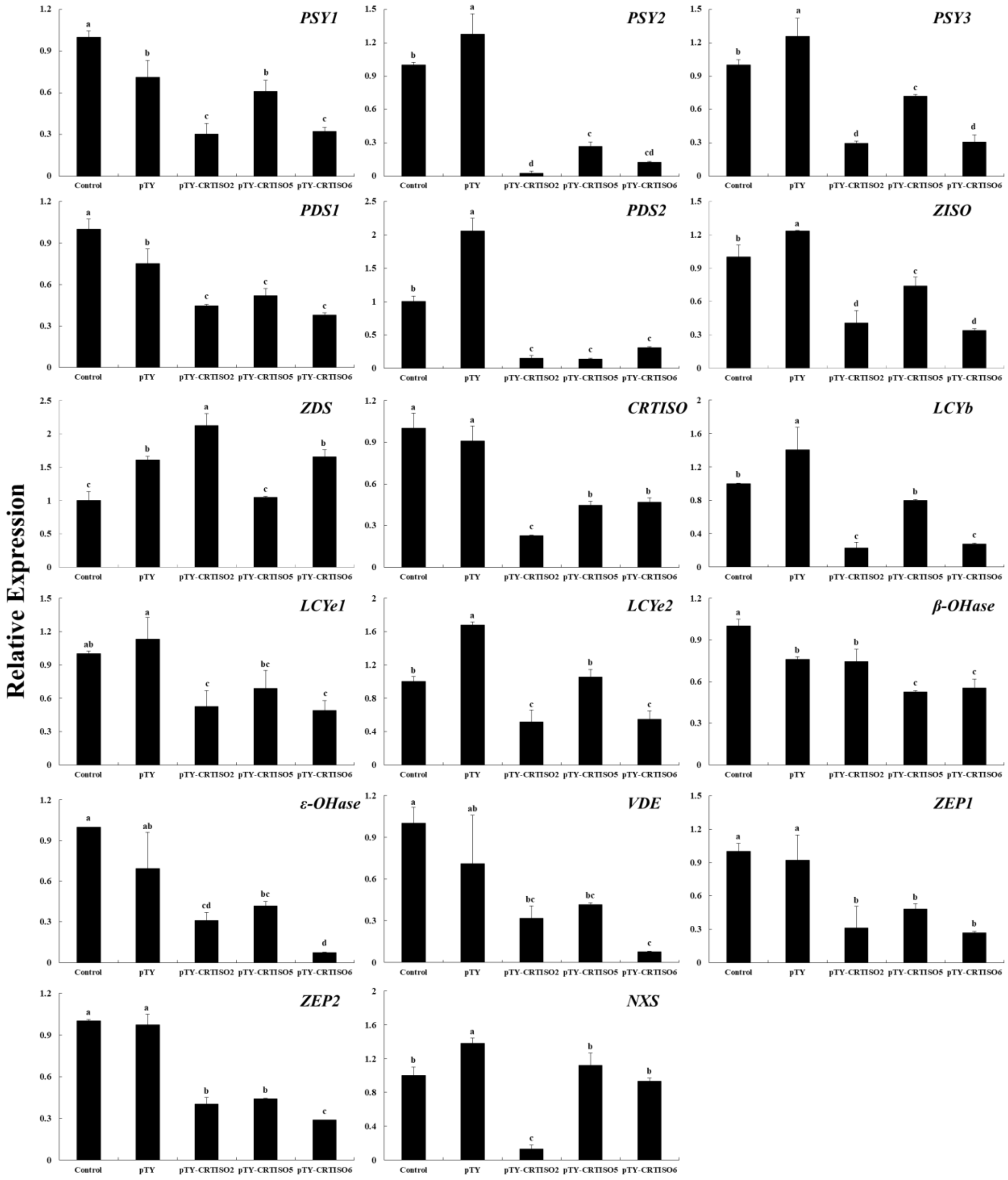

Supplementary Figure 1 Expressions of carotenoid biosynthetic genes in control, pTY and pTY-BoaCRTISO plants. Samples of leaves were taken from control, pTY, and pTY-BoaCRTISO plants at one week after the last infiltration. The carotenoid biosynthetic gene expressions were calculated based on the respective expression of the respective genes in control plant. Data are expressed as mean ± standard deviation. The same letter in the same histogram indicates that there is no significant difference between the values tested by LSD ($p < 0.05$).

Supplementary Figure 2

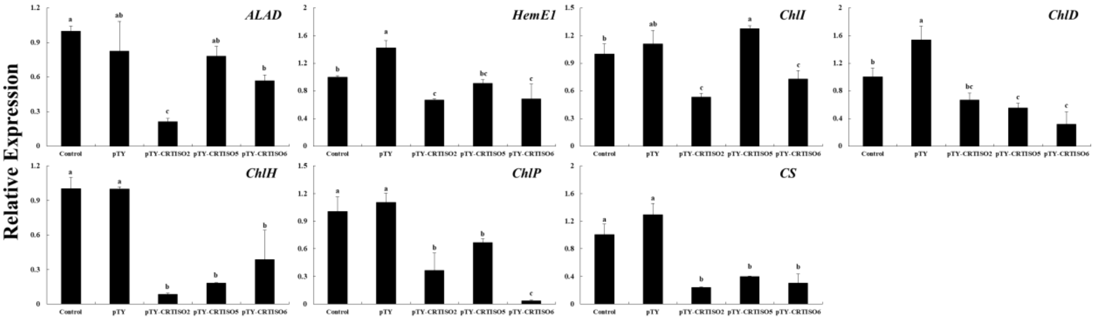

Supplementary Figure 2 Expressions of chlorophyll biosynthetic genes in control, pTY and pTY-BoaCRTISO plants. Samples of leaves were taken from control, pTY, and pTY-BoaCRTISO plants at one week after the last infiltration. The chlorophyll biosynthetic gene expressions were calculated based on the respective expression of the respective genes in control plant. Data are expressed as mean ± standard deviation. The same letter in the same histogram indicates that there is no significant difference between the values tested by LSD ($p < 0.05$). Abbreviation: ALAD, 5-aminolevulinic acid dehydratase; HemE1, glutamyl tRNA reductase; Chl, magnesium-chelatase; CS, chlorophyll synthase.

Supplementary Table 1 Primers used in this study

Primer name	Primer sequence (5'-3')	Aims
CRTISO-F	ATGAATCTCTGTCTCCGCAACCC	Molecular cloning of *CRTISO* gene and promoter
CRTISO-R	CTATGCGAGTGTCCTTAACCAACCA	
CRTISO promoter-F	ATGCACTGAAGGTGATATTCATGCTTGC	
CRTISO promoter-R	GCGGTGTTCCATCAAATCACAACC	
CRTISO-qPCR-F	AGATTGGGAGGGACTCACTCCA	Detection of gene expression
CRTISO-qPCR-R	CGTTCCCTTGTCCCGAGCAA	
β-actin-qPCR-F	CCAGAGGTCTTGTTCCAGCCATC	
β-actin-qPCR-R	GTTCCACCACTGAGCACAATGTTAC	
PSY1-qPCR-F	AAGGGCTGTAGAGTCTTCTAGA	
PSY1-qPCR-R	CGTTTTTGTTGTTTGCTTCCTC	
PSY2-qPCR-F	GGGACCTTGATGATCTGAAGAA	
PSY2-qPCR-R	TTCACCCAACAAACTCAAACTC	
PSY3-qPCR-F	TTCATGAGACTGCAGCTTAAGA	
PSY3-qPCR-R	CCCAACATAAGCTCTCTTGGTA	
PDS1-qPCR-F	GATCTCTTCACAAGCGCTTAAG	
PDS1-qPCR-R	GCTTCCAAGAAATTGACAGTGT	
PDS2-qPCR-F	TCATCTGGAGGTTGTGATTTGA	
PDS2-qPCR-R	ATATCCACACAAACTACCTGCA	
ZDS-qPCR-F	CGTTCCCTTGTCCCGAGCAA	
ZDS-qPCR-R	CCTCGGAGGTTTCATGTTAGGTCTTC	
ZISO-qPCR-F	CGTTGTTTGGATCGATAACTCC	
ZISO-qPCR-R	GCTAATCCACTATGCACAGTTG	
LCYb-qPCR-F	GTTGTTGATCTAGCTATCGTTGGC	
LCYb-qPCR-R	GAGTTTGGGGGAAGGATCGAT	
LCYe1-qPCR-F	GGTTTGTGTAGTAGAGTCGTCA	
LCYe1-qPCR-R	TCAACGAGCTTAGACTGTTCAT	
LCYe2-qPCR-F	CAGGTTCCGGTATAGAGAGTTG	
LCYe2-qPCR-R	ACGTATAGAATCTCCGAACCAC	
β-OHase-qPCR-F	CCTAATGGAGTGAAAAGCATCG	
β-OHase-qPCR-R	TCTTTACTGTTGATGGGAAGCT	
ε-OHase-qPCR-F	TCTCCTAAACCCAGATTCGTCTCC	
ε-OHase-qPCR-R	GCGAGTGAGTGATGTGAGCCA	
VDE-qPCR-F	AAGGCATTTCCTCACATCCTTA	
VDE-qPCR-R	ACCAAGAAAGTGCCTTTGATTC	
ZEP1-qPCR-F	AGAAGTCCTAGTTTCACTTGGG	
ZEP1-qPCR-R	TCTGTTTCCATGCTTGTTCAAG	
ZEP2-qPCR-F	AGAACTCCTAGTTTCACTTGGG	
ZEP2-qPCR-R	TATCCTCAATGGCCATACATCC	
NXS-qPCR-F	CAGTTGACATGCCAGCAAGTCC	
NXS-qPCR-R	CGAATCGGATGATACACTGGGGA	
ALAD-qPCR-F	AGCTTCCCATCGATCAAAGTAA	
ALAD-qPCR-R	GAAGTTGCAACTGGAAGAGAAG	

HemE1-qPCR-F	ATGTTACTCCTCAGGCTTATCG	
HemE1-qPCR-R	AGAGTTAGTGTGTCTTCTTGGG	
ChlI-qPCR-F	TCTCTTCTTCTTCCTCAACACC	
ChlI-qPCR-R	CCTTATTTGGATTCCTGCGTTT	
ChlD-qPCR-F	TCTCAACATATCGTCTCTTCCG	
ChlD-qPCR-R	GGTGATTCGAAGATAGCATTCG	
ChlH-qPCR-F	CACTACCACCAAACACTCATTC	
ChlH-qPCR-R	GAGACAGAGGACTTCACTTTGA	
ChlP-qPCR-F	TGGCGACTTTTACACTCAAATC	
ChlP-qPCR-R	ATCTTTCGCTCGATGAGGATC	
CS-qPCR-F	GGCGGAGACTGATACAGATAAA	
CS-qPCR-R	TACCAAGAAGCTGGTTAATGCT	
CRTISO GFP-F	CGGGATCCATGAATCTCTGTCTCCGCAACCC	Detection of subcellular localization (vector)
CRTISO GFP-R	GGACTAGTTGCGAGTGTCCTTAACCAACCA	
CRTISO VIGS-80nt	TGATAACTCAGGCGTTGAAGGCAGTTGGTCGTG AGATGGATCCATCTCACGACCAACTGCCTTCAA CGCCTGAGTTATCA	pTY-BoaCRTISO vector construction
pTY-CP-F	TCCACCCTCACCACCTTC	Detection of pTY-BoaCRTISO vector
pTY-CP-R	GGGACAGACCTCGCTAACT	

Supplementary Table 2 The expression level of *BoaCRTISO* under different exogenous treatments

Groups		Time							
		0 h	1 h	3 h	6 h	12 h	24 h	48 h	72 h
1	Control	1.05±0.36 a	1.39±0.32 a	1.77±0.25 a	1.30±0.13 b	1.21±0.23 b	0.39±0.04b	0.68±0.17 b	0.53±0.09 c
	Red light	1.05±0.36 a	1.01±0.15 b	1.05±0.10 b	1.04±0.08 b	0.99±0.08 b	0.33±0.00 b	0.58±0.05 b	0.71±0.01 b
	Blue light	1.05±0.36 a	1.13±0.04 ab	1.48±0.11 a	0.88±0.02 b	1.30±0.07 b	0.34±0.01 b	0.54±0.03 b	0.73±0.04 b
	Red and blue light	1.00±0.08 a	1.01±0.06 b	1.69±0.13 a	3.58±0.61 a	2.98±0.27 a	1.91±0.17 a	2.39±0.27 a	1.94±0.03 a
2	Control	1.05±0.36 a	1.39±0.32 a	1.77±0.25 a	1.30±0.13 b	1.21±0.23 c	0.39±0.04 b	0.68±0.17 c	0.53±0.09 b
	Dark	1.05±0.36 a	0.39±0.10 b	0.39±0.02 c	0.36±0.01 c	0.18±0.02 d	0.28±0.02 b	0.22±0.03 d	0.12±0.01 c
	Weak light	1.00±0.10 a	1.29±0.02 a	1.87±0.16 a	1.17±0.18 b	1.70±0.16 b	0.53±0.03 b	1.39±0.13 a	0.47±0.03 b
	Strong light	1.00±0.08 a	1.35±0.30 a	1.26±0.07 b	2.29±0.32 a	2.85±0.12 a	3.18±0.41 a	0.95±0.01 b	0.67±0.03 a
3	Control	1.00±0.08 a	1.10±0.11 a	1.76±0.04 a	3.66±0.21 a	3.76±0.12 a	1.92±0.61 a	2.02±0.08 b	1.31±0.04 b
	ABA	1.00±0.08 a	0.11±0.02 b	0.64±0.03 b	2.46±0.10 b	1.75±0.32 b	2.19±0.14 a	2.97±0.28 a	4.1±0.11 a
4	Control	1.00±0.08 a	1.10±0.05 b	1.76±0.04 b	3.66±0.21 b	3.76±0.12 b	1.92±0.61 a	2.02±0.08 a	1.31±0.04 b
	GA	1.00±0.08 a	3.57±0.27 a	2.02±0.09 a	5.69±0.08 a	4.69±0.15 a	1.12±0.07 a	2.01±0.10 a	2.42±0.13 a
5	Control	1.00±0.08 a	1.10±0.05 a	1.76±0.04 b	3.66±0.21 a	3.76±0.12 a	1.92±0.61 a	2.02±0.08 b	1.31±0.04 b
	MeJA	1.00±0.08 a	1.11±0.17 a	2.43±0.07 a	3.64±0.04 a	1.88±0.18 b	1.12±0.05 a	4.16±0.45 a	2.44±0.18 a

Data are expressed as mean ± SD. According to the least significant (LSD) test, the same letter in the column of the same group means that there is no significant difference between the values ($p<0.05$).
